# Supplementary material for: Targeting OLFML3 in Colorectal Cancer Suppresses Tumor Growth and Angiogenesis, and Increases the Efficacy of Anti-PD1 Based Immunotherapy
Source: Cancers (Basel). 2021 Sep 15;13(18):4625. doi: 10.3390/cancers13184625 (PMC8464773; doi:10.3390/cancers13184625)
Supplement: Supplementary file 1 [file cancers-13-04625-s001.zip › Figures and Supplementary documents/Supplementary Figure legends.pdf]

Article

Targeting OLFML3 in Colorectal Cancer Suppresses Tumor Growth and Angiogenesis, and Increases the Efficacy of Anti-PD1 Based Immunotherapy

Jimmy Stalin [1,2,*], Beat A. Imhof [1,3,†], Oriana Coquoz [2], Rachel Jeitziner [4], Philippe Hammel [1], Thomas A. McKee [5], Stephane Jemelin [1], Marine Poittevin [1], Marc Pocard [6,7], Thomas Matthes [8,9], Rachid Kaci [6,10], Mauro Delorenzi [4,11], Curzio Rüegg [2,†] and Marijana Miljkovic-Licina [1,8,9,†]

Supplementary figure legends:

Figure S1. Expression of OLFML3 in human tumors, as assessed by immunohistochemistry (IHC). (A) Intensity of vascular OLFML3 expression is ranked as follows: none (-), low (+), moderate (++), or strong (+++). (B) Representative microscopy images of OLFML3 immunostaining of esophageal adenocarcinoma and normal esophagus. (C) Representative microscopy images of OLFML3 immunostaining of papillary cell kidney carcinoma and normal kidney. (D) Representative microscopy images of OLFML3 immunostaining of prostate adenocarcinoma and normal prostate. (E) Representative microscopy images of immunostaining of OLFML3$^+$ of lung carcinoma and normal lung. (F) Representative microscopy images of OLFML3 immunostaining of uterus carcinoma (upper micrograph) and normal uterus (lower micrograph). OLFML3 staining is prominent in vessels. OLFML3 immunostaining is brown. All tissues are counterstained with hematoxylin-eosin. Scale bars, 200 μm (left) and 50 μm (right).

Figure S2. Expression of *OLFML3* mRNA is higher in stage 2–4 CRC than in stage 1, and correlates with expression of mRNA encoding angiogenesis-associated factors. (A) Expression of *OLFML3* mRNA in tumor samples from CRC patients, according to cancer stage. (B) Table summarizing the correlation between *OLFML3* mRNA expression and that of tumor-associated pro-angiogenic markers in CRC tumors. Expression of *OLFML3* in various subgroups of patients was compared using t-tests with equal variance. Expression of genes of interest was analyzed using Pearson's and Spearman's rank correlation analyses. (C) Forest plot showing the results of a meta-analysis of the association between *OLFML3* mRNA expression and that of tumor-associated angiogenic molecules in CRC tumor samples. All markers, except *VEGF-A*, exhibited a positive association (according to the odds ratio, OR) and a positive correlation with *OLFML3* mRNA expression. (D) Table summarizing the correlation between *OLFML3* mRNA expression and that of tumor-associated anti-angiogenic markers in CRC tumor samples. Expression of *OLFML3* in various subgroups of patients was compared using t-tests with equal variance. Expression of genes of interest was analyzed using Pearson's and Spearman's rank correlation analyses. (E) Table summarizing the correlation between expression of *OLFML3* mRNA and that of tumor-associated pro-lymphangiogenic markers in CRC tumor samples. Expression of *OLFML3* in various subgroups of patients was compared using t-tests with equal variance. Expression of genes of interest was analyzed using Pearson's and Spearman's rank correlation analyses.

Figure S3: Inhibition of colon cancer growth by an anti-Vegfr-2 antibody and correlation between OLFML3 and VEGF-A expression in colon cancer *in vivo*. (A) Growth of MC38 tumors (left) and *ex vivo* tumor size at sacrifice (right) after treatment with an anti-mouse Vegfr-2 blocking antibody (DC101) or isotype-matched rat IgG mAb (n = 6 mice per group). (B) Scatter plot with linear regression lines (black) showing the correlation between NanoString mRNA expression profiles of *Olfml3* and transcripts encoding human angiogenic factor *VEGF-A* in xenografted human DLD1 tumor extracts (n = 12), as determined by Pearson's and Spearman's rank correlation analyses.

Figure S4: Generation and characterization of rat anti-OLFML3 and recombinant anti-OLFML3 antibodies. (A) ELISA-based binding analysis of rat anti-OLFML3 mAbs (9F8, 46A9) to OLFML3 peptide A (pep A), OLFML3 peptide B (pep B), human recombinant OLFML3-FLAG-tagged (rOLFML3-FLAG), and human recombinant JAM-C-FLAG-tagged (rJAMC-FLAG) proteins. Isotype-matched rat IgG was used as a control (ctrl). (B) Schematic illustration of the rat (9F8/46A9,

left) and recombinant (rec9F8/rec46A9, right) mAbs, in which the Fc portion of the two rat mAbs (rat Fc) were replaced with mouse IgG2a Fc (mouse Fc). (C) ELISA dilution curves of OLFML3 peptide B detected by rec9F8 or rec46A9. (D) Competitive binding ELISA assays using OLFML3 peptide B to determine the binding specificity of rec9F8 (upper panel) and rec46A9 (lower panel). Upper panel: ELISA wells coated with 2.5 μg/mL OLFML3 peptide B were incubated with 1 μg/mL rec46A9, followed by incubation with increasing concentrations of rec9F8. Lower panel: ELISA wells coated with 2.5 μg/ml OLFML3 peptide B were incubated with 1 μg/mL rec9F8 antibody followed by incubation with increasing concentrations of rec46A9 antibody. Three experiments were performed, each in triplicate.

Figure S5. Anti-tumor effects of rat and recombinant OLFML3 antibodies on lung tumors. (A) Upper scheme: experimental protocol of Lewis lung carcinoma 1 (LLC1) cell injection into C57BL/6J mice at day 1 and antibody treatments at days 4 and 7. Macroscopic view (lower left) and weight (lower right) of LLC1 tumors at sacrifice excised from mice treated with either rat anti-OLFML3 mAbs (9F8, 46A9) or control IgG as indicated. (B) Macroscopic view (left) and weight (right) of LLC1 tumors at sacrifice excised from mice treated with recombinant anti-OLFML3 (rec46A9) or control IgG as indicated. (C) Scheme showing the experimental protocol of Lewis lung carcinoma 1 (LLC1) cell injection into C57BL/6J mice at day 1 and mice treatments at days 5, 7 and 9 with 46A9 mAb. (D) Tumor volume measured daily using a caliper from day 5 to sacrifice in mice treated with increasing doses of 46A9 mAb (i.e., 30, 150, and 750 μg/mouse/injection). (E) Upper scheme: experimental protocol of Lewis lung carcinoma 1 (LLC1) cell injection into C57BL/6J mice at day 1 and antibody treatments at days 5, 7 and 9 with 46A9, 9F8, or control IgG (11G8). Below: Tumor volume measured daily using a caliper from day 5 to sacrifice in mice treated with 46A9, 9F8, or control IgG (11G8). Results represent the mean value ± SEM; $*P < 0.05$; $**P < 0.01$; $***P < 0.01$.

Figure S6. Anti-OLFML3 antibody treatment decreases vascularization, pericyte coverage, and expression of human tumor-derived PLGF, but does not affect other pro-angiogenesis factors. (A) Representative confocal images comparing co-cultures of HUVECs (red) and human brain pericytes (green) for their ability to form endothelial capillary-like networks in Matrigel in the presence of rec9F8 mAb or a $IgG2_a$ isotype control antibody (Ctrl IgG) at 1, 3, 5, and 7h after seeding (upper panel). Quantification of total branch length of capillary-like networks formed by HUVEC and pericyte co-cultures (lower panel). Results represent the mean value ± SEM (three experiments; each group tested in triplicate); $*P < 0.05$; $**P < 0.01$. (B) *OLFML3* mRNA levels in human vascular (HUVEC), mouse vascular (bEnd.5), and mouse lymphatic (LyEnd.1) endothelial cell lines, and in primary mouse pericytes (Pericytes), as determined by qRT-PCR. Average C_T was calculated for both *OLFML3* and *EEF1*; $\Delta C_T = (C_{T,\ OLFML3} - C_{T,\ EEF1})$. Data are representative of three independent experiments. Bars represent the mean ± SEM. (C) Experimental design for gene expression profiling of angiogenesis-associated molecules in tumor extracts isolated from human DLD1-bearing mice treated with rec9F8, bevacizumab, or IgG control mAbs (n = 6–8 mice per group). To identify both stromal cell (mouse, see Figure 4 in main text) and tumor (human) transcript signatures, we used NanoString mRNA arrays with both human and mouse-specific mRNA probes. (D) Human mRNA expression profiling, using NanoString arrays, of angiogenic factors in DLD1 tumor extracts from mice treated with rec9F8, bevacizumab, or control IgG mAbs. Results are normalized against the values for controls. Bars represent the mean ± SEM (three experiments); $*P < 0.05$.

Figure S7. Gating strategies used for flow cytometry to quantify tumor-associated macrophages and pro-inflammatory macrophages in MC38 tumors isolated from tumor-bearing mAb-treated mice. (A) Flow cytometry gating strategies used for $CD45^+$ cells, macrophages ($CD45^+/CD11b^+/F4\text{-}80^+$), and TAMs ($CD45^+/CD11b^+/CD68^+/F4\text{-}80^+$). (B) Quantification of pro-inflammatory M1 TAMs ($CD45^+/MHC2^+/Ly6C^+$) in MC38 tumor-bearing mice treated with rec9F8 or control IgG, and in OLFML3 knock-out versus WT mice. (C) Representative flow cytometry gating profiles for $CD45^+/CD11b^+/F4\text{-}80^+$ and $CD45^+/CD11b^+/CD68^+/F4\text{-}80^+$ macrophages from MC38 tumors extracted from mice treated with clodronate liposomes alone, or with clodronate liposomes plus rec9F8, as well as the corresponding controls (control liposomes, lipo ctrl; control IgG mAb, Ctrl IgG). (D). Quantification of pro-inflammatory M1 TAMs ($CD45^+/MHC2^+/Ly6C^+$) in MC38 tumor-bearing mice treated with either control liposomes or clodronate liposomes, either alone or in combination with rec9F8 or control antibodies. (E) Forest plot showing the results of a meta-analysis of the association between *OLFML3* mRNA expression and that of certain tumor-associated macrophage markers in CRC tumor samples. Markers exhibited a positive association (using the odds ratio, OR) and positive correlation with *OLFML3* mRNA expression. (F) MC38 tumor growth curve (left) and *ex vivo* volume at the end of the experiment (right); tumors were isolated from mice treated with rec46A9M, rec46A9, or control IgG mAbs (n = 7 per group). Bars (right) represent the mean ± SEM; $*P < 0.05$.

(G) Quantification of Pecam1-positive events obtained by PECAM1 immunostaining of the tumors from (D).

Figure S8. Anti-OLFML3 antibody treatment promotes recruitment of NKT innate immune cells, but not B and T lymphocytes, into colorectal tumors. (A) Flow cytometry quantification of tumor-infiltrating $CD4^+$ T lymphocytes ($CD45^+/CD3^+/CD4^+$) and $CD8^+$ T lymphocytes ($CD45^+/CD3^+/CD8^+$) isolated from mice bearing MC38 tumors treated with rec9F8, or in tumors from Olfml3-deficient mice. (B) Flow cytometry quantification of tumor-infiltrating NKT cells ($CD45^+/CD3^+/NK1.1^+$) isolated from wild-type (WT) or *Olfml3* knock-out mice bearing MC38 tumors treated with rec9F8 or clodronate. Right panel: mice treated with control IgG, rec9F8, clodronate (Clo), or control liposomes (Lipo Ctrl), either alone or in combination. Left panel: Olfml3-deficient (KO) and WT mice treated with rec9F8 or control IgG (Ctrl IgG). Bars represent the mean ± SEM; $^*P < 0.05$. (C) Forest plot showing the results of a meta-analysis of the association between *OLFML3* mRNA expression and that of immune cell markers and immune checkpoint inhibitors in CRC patient-derived tumor samples. Markers exhibited a positive association (as assessed by the odds ratio, OR) and positive correlation with *OLFML3* mRNA expression. (D) Scatter plot with linear regression lines comparing expression of *CTLA4* (up) and *HAVCR2* (down) mRNA with that of *OLFML3* mRNA in tumor samples from CRC patients. Differences between two groups were evaluated using Pearson's correlation and Spearman's rank correlation analyses.
