# Supplementary material for: Targeting OLFML3 in Colorectal Cancer Suppresses Tumor Growth and Angiogenesis, and Increases the Efficacy of Anti-PD1 Based Immunotherapy
Source: Cancers (Basel). 2021 Sep 15;13(18):4625. doi: 10.3390/cancers13184625 (PMC8464773; doi:10.3390/cancers13184625)

Supplementary Fig. 1

A

	Tissue	Vascular expression intensity
1	Esophagus adenocarcinoma	-
	Normal esophagus	-
2	Papillary cell carcinoma	+/-
	Normal kidney	+/-
3	Prostate adenocarcinoma	++
	Normal prostate	+
4	Lung adenocarcinoma	++
	Normal lung	+
5	Uterus carcinoma	+++
	Normal uterus	+/-

B

Esophagus adenocarcinoma

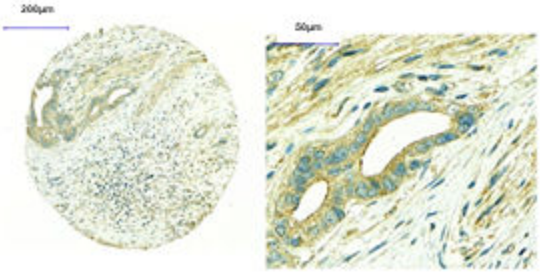

Normal esophagus

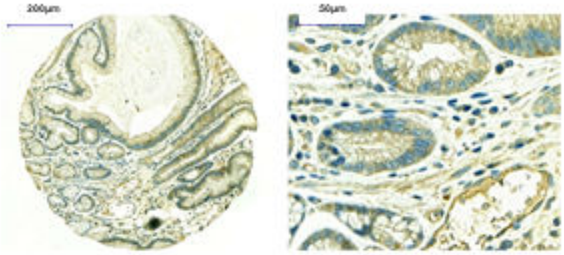

C

Papillary cell carcinoma

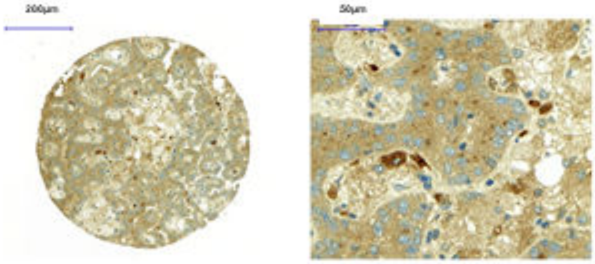

Normal kidney

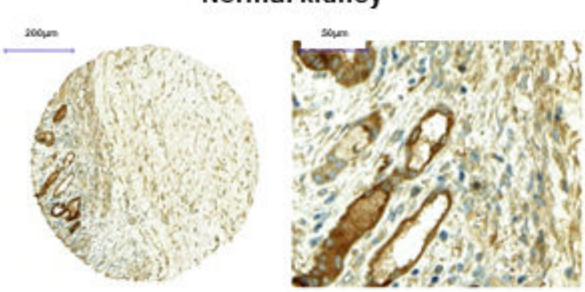

D

Prostate adenocarcinoma

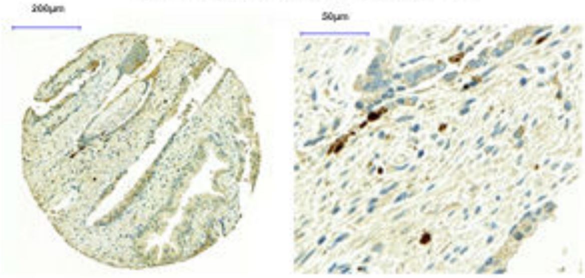

Normal prostate

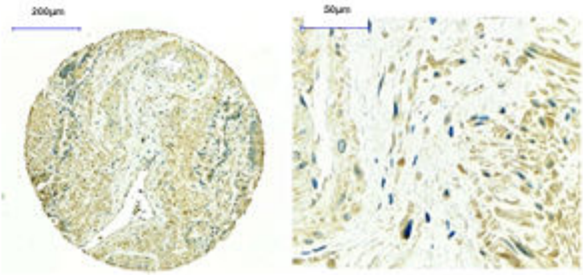

E

Lung adenocarcinoma

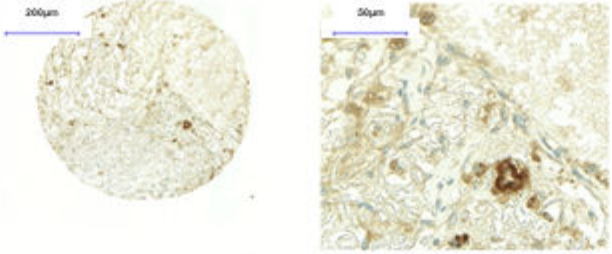

Normal lung

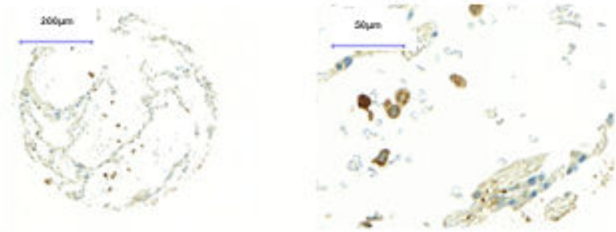

F

Uterus carcinoma

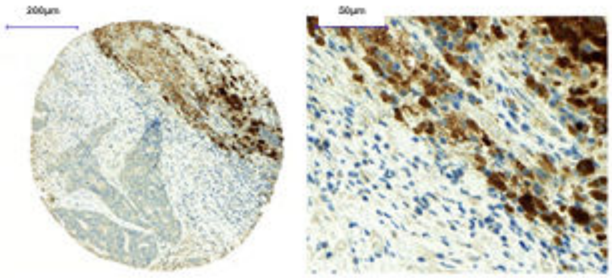

Normal uterus

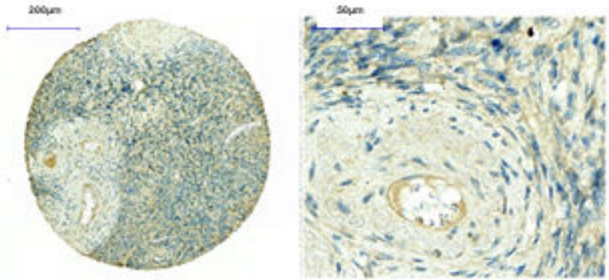

Supplement: Supplementary file 1 [file cancers-13-04625-s001.zip › Figures and Supplementary documents/Supplementary Figures/Figure S1.pdf]
