# Supplementary material for: Targeting OLFML3 in Colorectal Cancer Suppresses Tumor Growth and Angiogenesis, and Increases the Efficacy of Anti-PD1 Based Immunotherapy
Source: Cancers (Basel). 2021 Sep 15;13(18):4625. doi: 10.3390/cancers13184625 (PMC8464773; doi:10.3390/cancers13184625)
Supplement: Supplementary file 1 [file cancers-13-04625-s001.zip › Figures and Supplementary documents/Supplementary Figures/Figure S2.pdf]

A

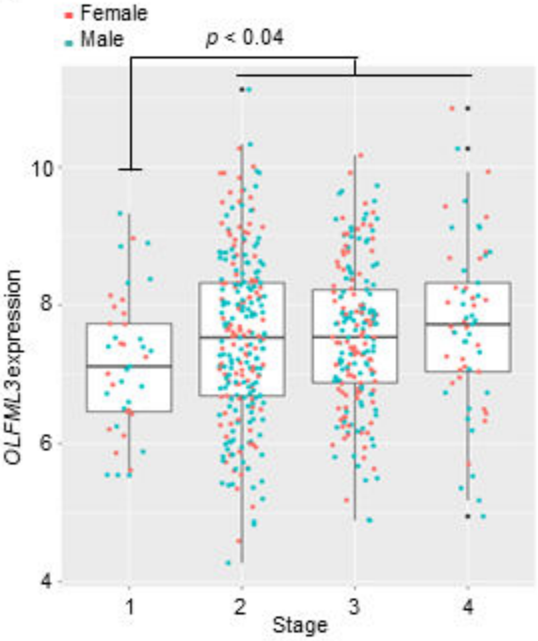

C

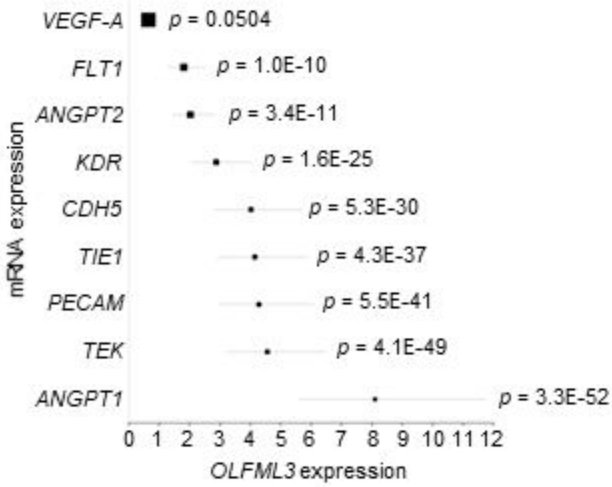

B

Name	Correlation score	*p* - value
ANGPT1	0.58	3.30E-52
KDR	0.42	1.56E-25
ANGPT2	0.27	3.4E-11
PECAM1	0.52	5.50E-41
TIE1	0.50	4.28E-37
CDH5	0.45	5.30E-30
TEK	0.56	4.10E-49
C3	0.54	1.10E-45
CX3CR1	0.53	1.90E-42
FGF1	0.52	1.20E-41
HGF	0.52	5.50E-41
PRKD1	0.56	2.20E-48
PTGIS	0.55	1.80E-46
RHOJ	0.52	1.30E-41
FLT1	0.26	1.04E-10
VEGFA	-0.08	0.0504

D

Name	Correlation score	*p* - value
CREB3L1	-0.15	0.0004
EPHA2	-0.13	0.0016
E2F2	-0.28	6.49E-12
KLF4	-0.14	0.0007
EFNA3	-0.085	0.04
LIF	-0.082	0.048

E

Name	Correlation score	*p* - value
FLT4	0.45	4.89E-30
PDPN	0.52	1.12E-41

Supplementary Fig. 2
