# Supplementary material for: Targeting OLFML3 in Colorectal Cancer Suppresses Tumor Growth and Angiogenesis, and Increases the Efficacy of Anti-PD1 Based Immunotherapy
Source: Cancers (Basel). 2021 Sep 15;13(18):4625. doi: 10.3390/cancers13184625 (PMC8464773; doi:10.3390/cancers13184625)
Supplement: Supplementary file 1 [file cancers-13-04625-s001.zip › Figures and Supplementary documents/Supplementary_informations.pdf]

Article

Targeting OLFML3 in Colorectal Cancer Suppresses Tumor Growth and Angiogenesis, and Increases the Efficacy of Anti-PD1 Based Immunotherapy

Jimmy Stalin [1,2,*], Beat A. Imhof [1,3,†], Oriana Coquoz [2], Rachel Jeitziner [4], Philippe Hammel [1], Thomas A. McKee [5], Stephane Jemelin [1], Marine Poittevin [1], Marc Pocard [6,7], Thomas Matthes [8,9], Rachid Kaci [6,10], Mauro Delorenzi [4,11], Curzio Rüegg [2,†] and Marijana Miljkovic-Licina [1,8,9,†]

Supplementary materials and methods:

Analysis of tumor growth in xenograft-bearing animals

Mouse and human tumor cell line xenografts were produced by subcutaneous injection of tumor cell suspensions in PBS (1×10^6 cells for LLC1; 5×10^5 cells for DLD1 and MC38) into the back (LLC1, MC38, and DLD1) of mice. When the tumors reached 40–50 mm^3, mice received a twice weekly intraperitoneal injection of mAbs at a dose of 100 µg per mouse (anti-OLFML3 antibodies), 100 µg per mouse (bevacizumab), 600 µg per mouse (DC101), 100 µg per mouse (Pali IgG2a, a control antibody for recombinant anti-OLFML3 antibodies), or 600 µg per mouse (rat IgG, a control antibody for DC101) until sacrifice.

Macrophages were depleted from tumors by administration of a bi-weekly intraperitoneal injection of clodronate-loaded liposomes. Control and clodronate-loaded liposomes were obtained from LIPOSOMA. Therapeutic monoclonal antibodies, including anti-mouse PD-1 (clone RMPI-14 IgG2a; 10 mg/kg) and anti-CSF1-receptor (clone AFS98), were obtained from BioXCell and administrated twice per week.

Tumor size was measured twice per week using a caliper, and tumor volume was determined according to the equation: length × width × thickness × 0.5236. Tumor size and mass were also evaluated after sacrifice. At the time of sacrifice, animals were anesthetized with a mix of ketamine and xylazine (80 mg/kg and 10 mg/kg, respectively) before intra-tracheal instillation of 10% formalin to fix the tissues. After paraffin embedding, samples were cut into sections at five distinct levels and stained with hematoxylin and eosin (H&E). Slides were scanned using an Axioscan automatic slide scanner (Zeiss, Plan-Apochromat 20×/0.8).

In vivo toxicity assay

LLC1 cells were xenografted subcutaneously into mice, which were then treated with the indicated doses (30, 150, or 750 µg/mouse) of anti-OLFML3 antibodies or with rat control IgG. Organs were removed, fixed in 4% paraffin in PBS, and sectioned using a cryostat. Tissue integrity was visualized after H&E staining.

Flow cytometry

After dissection, tumors were diced with razor blades and digested using a gentleMACS Dissociator and reagents from the corresponding mouse and human tumor dissociation kits (Miltenyi Biotech). Single tumor cell suspensions were obtained by straining through a 70 µm mesh filter, after which the strained cells were washed twice in FACS buffer (PBS/Fetal Calf Serum 5%/5 mM EDTA). Cells were incubated for 30 minutes at 4°C with an anti-CD16/CD32 Fc blocking antibody (BD Biosciences) to block Fc receptor binding and then washed once with FACS buffer. The cells were stained with the indicated fluorophore-conjugated antibodies and analyzed on a Gallios flow cytometer (Beckman Coulter) to quantify the proportion of blood vascular endothelial cells and lymphatic vascular endothelial cells in the tumors, as previously described [1-2].

ELISA

Platelet-diminished plasma was prepared from mouse blood using a two-step centrifugation method. Mouse Vegf-a and mouse Plgf were assayed using the corresponding DuoSet ELISA kits (R&D Systems). Mouse Vegf-c was assayed using a mouse VEGF-C ELISA kit (CusaBio).

Endothelial cell isolation and culture

Umbilical cords were collected for endothelial cell isolation. HUVECs were isolated as previously described [3]. Briefly, the cord vein was cleared by perfusion with PBS, followed by incubation with collagenase (1 mg/ml; Invitrogen, Carlsbad, California, United-States) in PBS for 15 minutes at 37°C. The vein was then perfused with PBS to remove the endothelial cells and the suspension was centrifuged at 200 × *g* for 5 minutes. The cell pellet was resuspended and maintained in complete M199 containing 10% fetal bovine serum (FBS), 15 μg/mL endothelial cell growth supplement (Upstate Biotechnology), 100 μg/mL heparin (Sigma-Aldrich), 50 μM hydrocortisone (Sigma-Aldrich), and 10 μg/mL vitamin C (Sigma-Aldrich). Cells were cultured and used up to passage 5.

Pericyte cell culture

Human brain pericytes were purchased from ScienCell Research Laboratories and cultured on 2 μg/cm^2 poly-L-lysine-coated culture vessels in pericyte medium supplemented with 2% FBS, 1% Pericyte Growth Supplement, and 1% penicillin-streptomycin solution (all from ScienCell Research Laboratories). Cells were cultured up to passage 7.

Tumor and immortalized cell line cultures

bEnd.5 mouse immortalized brain vascular endothelial cells, LyEnd.1 mouse immortalized lymphatic endothelial cells, LLC1 mouse lung cancer cells, DLD1 human colorectal cancer cells, and MC38 mouse colorectal cancer cells were cultured in DMEM (Life Technologies) supplemented with 10% FBS, 1% penicillin-streptomycin solution, 1% L-glutamine, and 1% sodium pyruvate. Cells were grown at 37°C in a humidified atmosphere containing 5% CO_2.

Endothelial–pericyte co-culture assay

The assay was adapted from a previous experimental protocol [4]. Briefly, adherent HUVECs and pericytes were washed in serum-free M199 medium and then incubated for 30 min at 37°C with either CellTracker Green CMFDA488 dye (#C7025; 1:1000 dilution; Life Technologies) or CellTracker Orange CMRA548 dye (#C-34551; 1:1000 dilution; Life Technologies) for labeling. *In vitro* angiogenesis μ-slides (Ibidi) were incubated on ice, and 10–12 μL of ice-cold growth factor-reduced Matrigel (Corning) was added to each well and allowed to polymerize. Subsequently, the complete M199 medium (50 μL) was added to each well and incubated for 45 min at 37°C. HUVECs (5,000 cells) and pericytes (2,500–5,000 cells) were added to the polymerized Matrigel and cultured in complete M199 medium (see above) in the presence of control (isotype) or anti-OLFML3 monoclonal antibodies (46A9 and 9F8) for 7–10 h. Live-cell time-lapse imaging was performed on a NikonA1R microscope. Z-stack 3D-reconstruction and quantification analysis of capillary-like networks were performed using Fiji/ImageJ with the Angiogenesis Analyzer toolset.

Immunohistochemistry

Frozen tumor tissue samples were processed for immunohistochemistry and stained as previously described [5]. Briefly, samples were incubated for 1 h at room temperature with rabbit anti-Olfml3^{A+B} serum (16), rat monoclonal anti-PECAM-1 [6], mouse monoclonal anti-α-SMA [7], or rabbit anti-NG2 (clone 132.38; Millipore). Vessel density was quantified using confocal images of human tumor sections stained with PECAM-1. Metamorph6.0 (Molecular Devices) was used for image analysis, and the ratio of the total pixel count for PECAM-1 to that of DAPI was measured and normalized against that of the

control. Quantification of $NG2^+$ or α-SMA^+ pericyte density was measured as a ratio of the total pixel count of NG2 or α-SMA to the total pixel count of PECAM-1, and normalized against that of the control. Control values were defined as 100%, and the relative values for anti-OLFML3-treated mice were calculated accordingly.

Quantitative reverse transcription-polymerase chain reaction (qRT-PCR)

Total RNA from tumor samples was extracted using the Nucleospin RNA Extraction Kit (Macherey-Nagel, Oensingen, Switzerland). Extracted total RNA was quantified by measuring the OD at 260 nm using a NanoDrop spectrophotometer (Thermo Fisher Scientific). Reverse transcription (RT) was then carried out with 1 µg total RNA in a reaction mixture containing RT buffer, dNTPs, random primer, MultiScribe reverse transcriptase, and H_2O to a final volume of 10 µl accordingly to the manufacturer's protocol (Applied Biosystems, California, United-States). The RT reaction was performed in a thermocycler as follows: 25°C for 10 min, 37°C for 2 h, and 85°C for 5 min, followed by a hold at 4°C. For RT-PCR, 50 ng/µl cDNA was added to 24 µl of a mixture consisting of 12 µl SYBR Green (Agilent/Stratagene, Santa Clara, California, United-states), forward and reverse primers, and H_2O. PCR was then carried out on a Stratagene Mx 3000 Pro QPCR device (Agilent Technologies) as follows: one cycle for 10 mins at 95°C, followed by 40 cycles for 30 seconds at 95°C and 1 minute at 60°C. The results were analyzed using the MxPro software (Stratagene). qRT-PCR data are presented as ΔCt values of the genes of interest relative to the housekeeping gene *EEF1*.

NanoString nCounter mRNA assay

The amount of mRNA content in tumor biopsies was determined using the nCounter platform (NanoString Technologies) and analyzed with nSolver Software on the iGE3 Genomics Platform of the University of Geneva.

Generation of monoclonal anti-OLFML3 antibodies

Anti-human/mouse OLFML3 rat monoclonal antibodies were generated by immunizing rats with a 13-aa peptide epitope (referred as OLFML3 peptide B in the main text) derived from the olfactomedin-like domain (Supplementary Fig. 3A). Hybridomas were produced by fusing antibody-producing rat B lymphocytes with mouse Sp2/0 myeloma cells. The antibody-producing hybridomas were cloned and screened for production of mAbs specific for human OLFML3 (Supplementary Fig. 3B). The mAb-producing hybridomas were assayed by ELISA to determine their affinities for OLFML3 peptide B and a recombinant human OLFML3-FLAG-tagged protein. Clones 9F8 and 46A9, both of which produced IgG2b anti-OLFML3 antibodies, were selected for further use.

The IgG genes expressed by the two rat hybridoma cells were cloned into the pCR2.1 (Invitrogen) by Fusion Antibodies (Belfast, UK), and the variable regions of the heavy and light Ig chains were sequenced. The sequences were transferred to Evitria (Schlieren, Switzerland), and recombinant antibodies containing mouse Fc of the IgG2b type were constructed by gene engineering. Antibodies were produced in mammalian cells and IgG-purified at the Evitria facilities.

Detection of OLFML3 antibody epitopes

MaxiSorp immunoplates (Nunc) were coated with 1 µg/mL of newly generated OLFML3 antibody. Wells were blocked with 1% bovine serum albumin (BSA) and incubated with recOLFML3-FLAG at 0.5 mg/mL in PBS containing 0.05% Tween-20 and 0.5% BSA. Next, biotinylated-M2 antibody (2 mg/mL; Sigma-Aldrich, Saint-Louis, United-States) specific for the FLAG peptide was added. Bound M2 was detected using streptavidin-HRP (Jackson ImmunoResearch Laboratories, Bar Harbor, United-States) and the Substrate Reagent Pack (R&D Systems, Minneapolis, United-States). OD (450 nm) was read on a kinetic microplate reader and data were analyzed using the SoftMax Pro software (Molecular Devices, San José, United-States).

Competitive ELISA

Microlon immunoplates with medium binding capacity (Microlon 200; Greiner) were coated with OLFML3 peptide B at 2.5 μg/mL, washed, and incubated with 1 μg/mL rec46A9, followed by incubation with increasing concentrations of 9F8 antibody. The second competitive ELISA assay was performed by binding immunoplate-coated OLFML3 peptide B to the 9F8 antibody (1 μg/mL), followed by incubation with increasing concentrations of rec46A9 mAb. MAb rec46A9 was visualized using anti-mouse IgG-HRP (#715-035-151; Jackson) with minimal cross-reactivity against rat IgG in the presence of 2% normal rat serum. The rat 9F8 antibody was visualized using an anti-rat IgG-HRP (#712-035-153; Jackson), which showed minimal cross-reactivity against mouse IgG in the presence of 2% normal mouse serum. Detection of primary antibodies was performed in triplicate. HRP conjugated secondary antibodies were visualized using the Substrate Reagent Pack. OD (450 nm) was read on a kinetic microplate reader and data were analyzed using the SoftMax Pro software.

Supplementary references:

1 Nowak-Sliwinska P, Alitalo K, Allen E, et al. Consensus guidelines for the use and interpretation of angiogenesis assays. Angiogenesis. 2018 Aug;21(3):425-532.

2 Stalin J, Garrido-Urbani S, Heitz F, et al. Inhibition of host NOX1 blocks tumor growth and enhances checkpoint inhibitor-based immunotherapy. Life Sci Alliance. 2019 Jun 27;2(4):e201800265.

3 Nachman RL, Jaffe EA. Endothelial cell culture: beginnings of modern vascular biology. J Clin Invest. 2004 Oct;114(8):1037-40.

4 Tigges U, Welser-Alves JV, Boroujerdi A, et al. Novel and simple method for culturing pericytes from mouse brain. Microvasc Res. 2012 Jul;84(1):74-80.

5 Miljkovic-Licina M, Hammel P, Garrido-Urbani S, et al. Sushi repeat protein X-linked 2, a novel mediator of angiogenesis. FASEB J. 2009 Dec;23(12):4105-16.

6 Piali L, Albelda SM, Baldwin HS, et al. Murine platelet endothelial cell adhesion molecule (PECAM-1)/CD31 modulates beta 2 integrins on lymphokine-activated killer cells. Eur J Immunol. 1993 Oct;23(10):2464-71.

7 Skalli O, Ropraz P, Trzeciak A, et al. A monoclonal antibody against alpha-smooth muscle actin: a new probe for smooth muscle differentiation. J Cell Biol. 1986 Dec;103(6 Pt 2):2787-96.
