# Supplementary figures and images for: Targeting OLFML3 in Colorectal Cancer Suppresses Tumor Growth and Angiogenesis, and Increases the Efficacy of Anti-PD1 Based Immunotherapy
Source: Cancers (Basel). 2021 Sep 15;13(18):4625. doi: 10.3390/cancers13184625 (PMC8464773; doi:10.3390/cancers13184625)

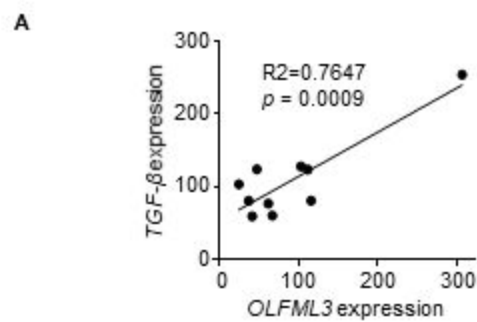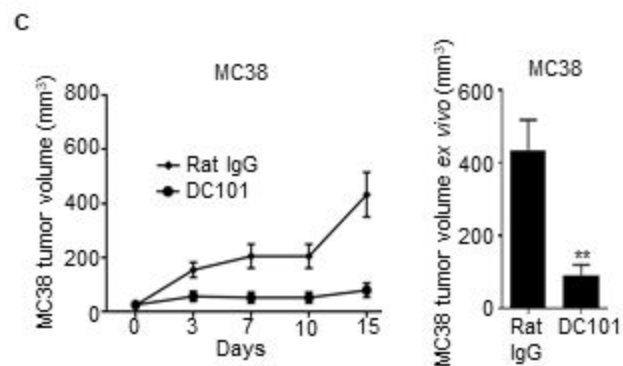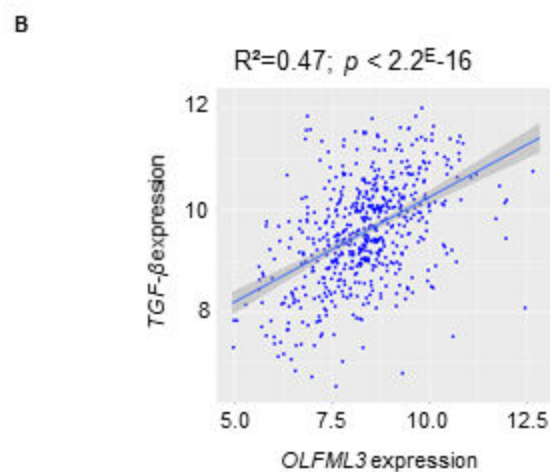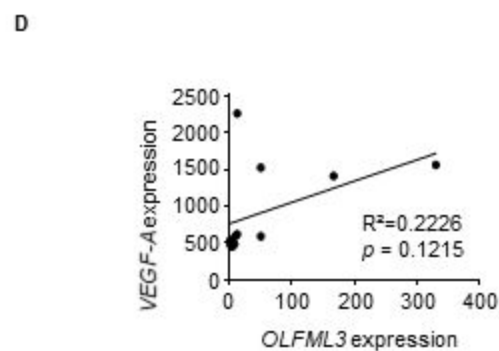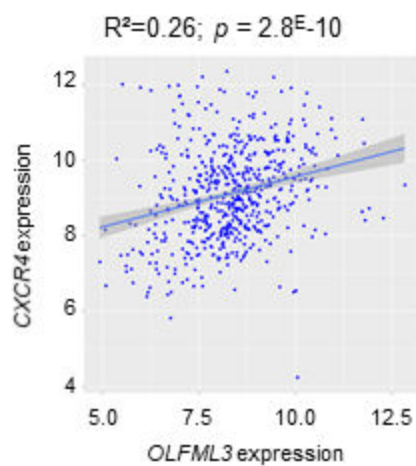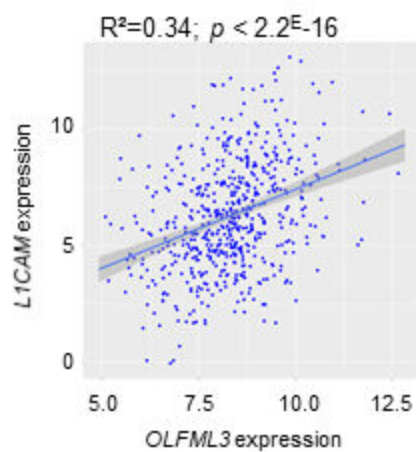

Supplement: Supplementary file 1 [file cancers-13-04625-s001.zip › Figures and Supplementary documents/Supplementary Figures/Figure S3.pdf]

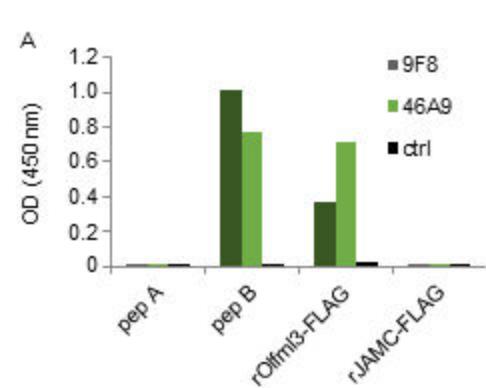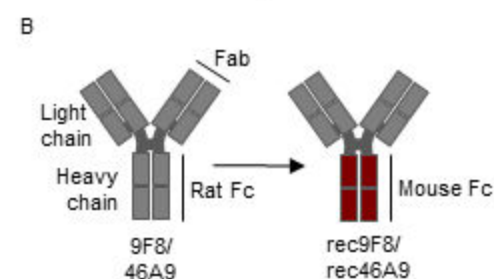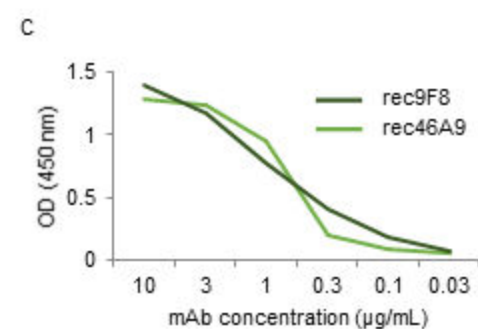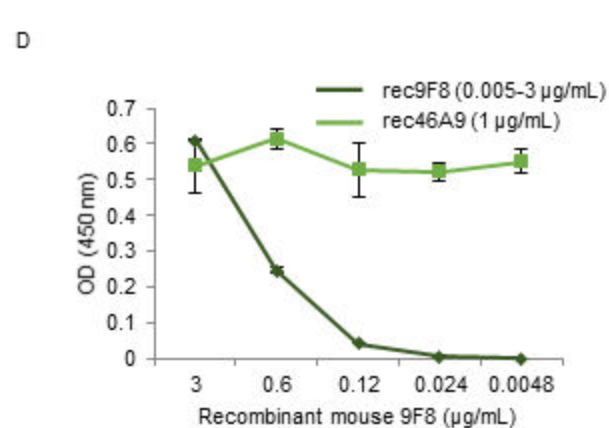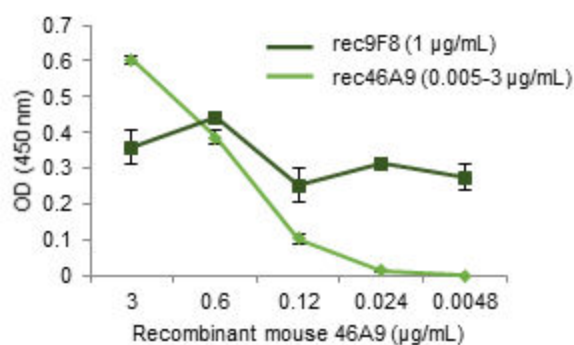

Supplementary Fig. 4

Supplement: Supplementary file 1 [file cancers-13-04625-s001.zip › Figures and Supplementary documents/Supplementary Figures/Figure S4.pdf]

A

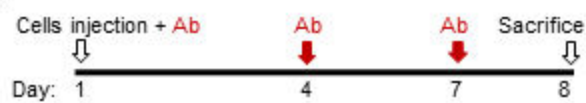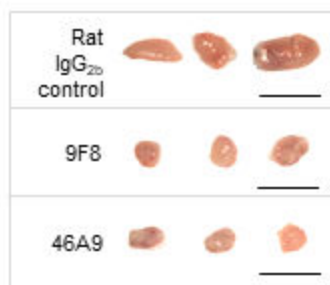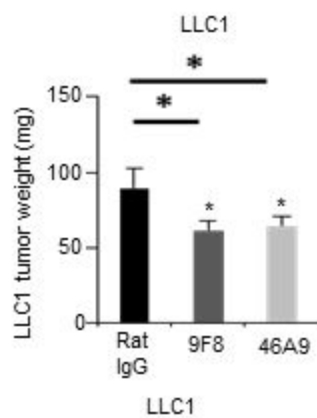

B

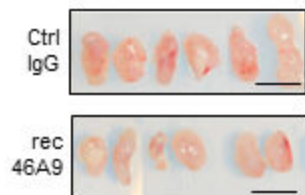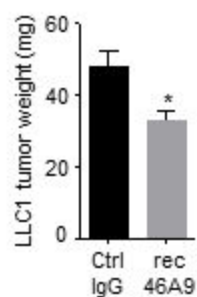

C

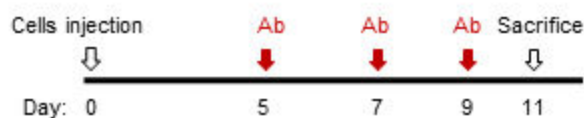

D

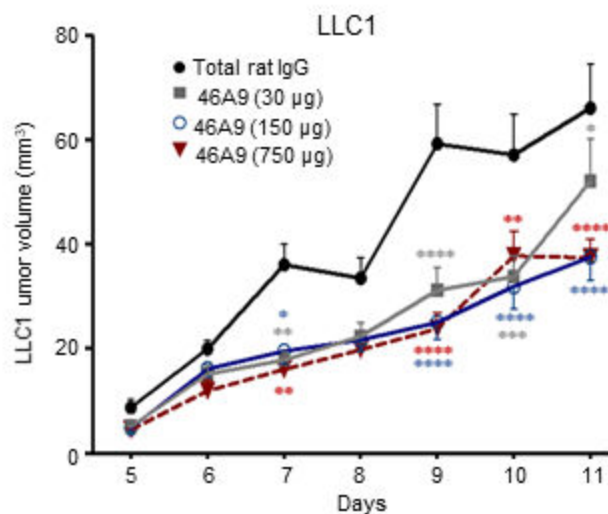

E

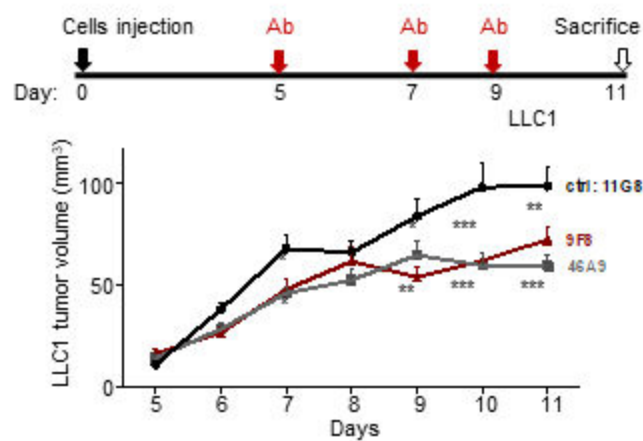

Supplementary Fig. 5

Supplement: Supplementary file 1 [file cancers-13-04625-s001.zip › Figures and Supplementary documents/Supplementary Figures/Figure S5.pdf]

A

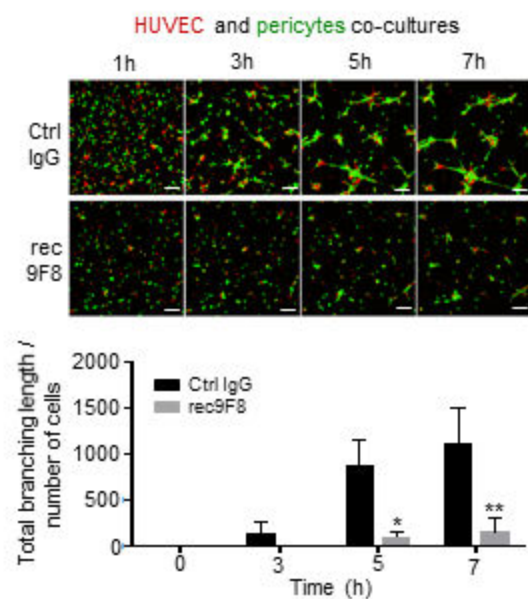

B

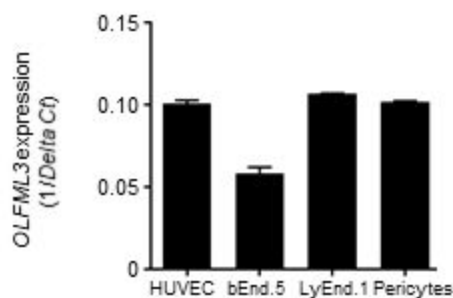

C

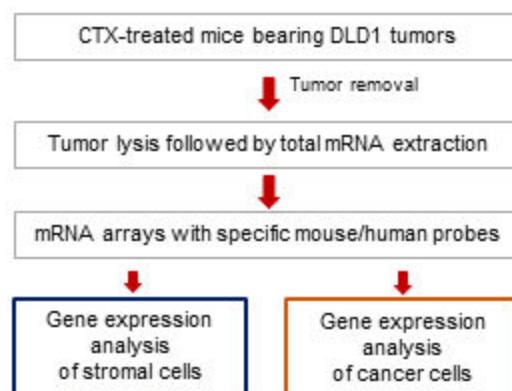

D

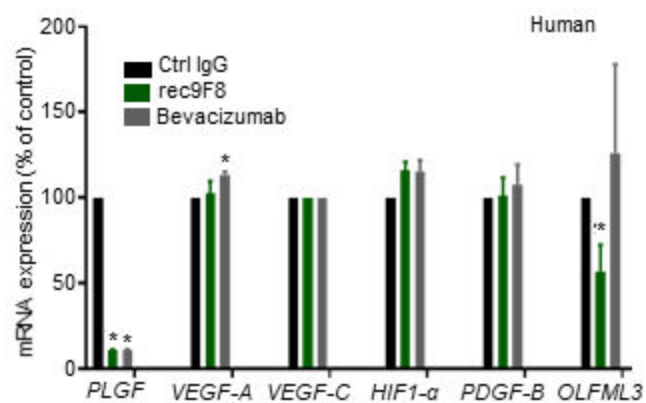

Supplementary Fig. 6

Supplement: Supplementary file 1 [file cancers-13-04625-s001.zip › Figures and Supplementary documents/Supplementary Figures/Figure S6.pdf]

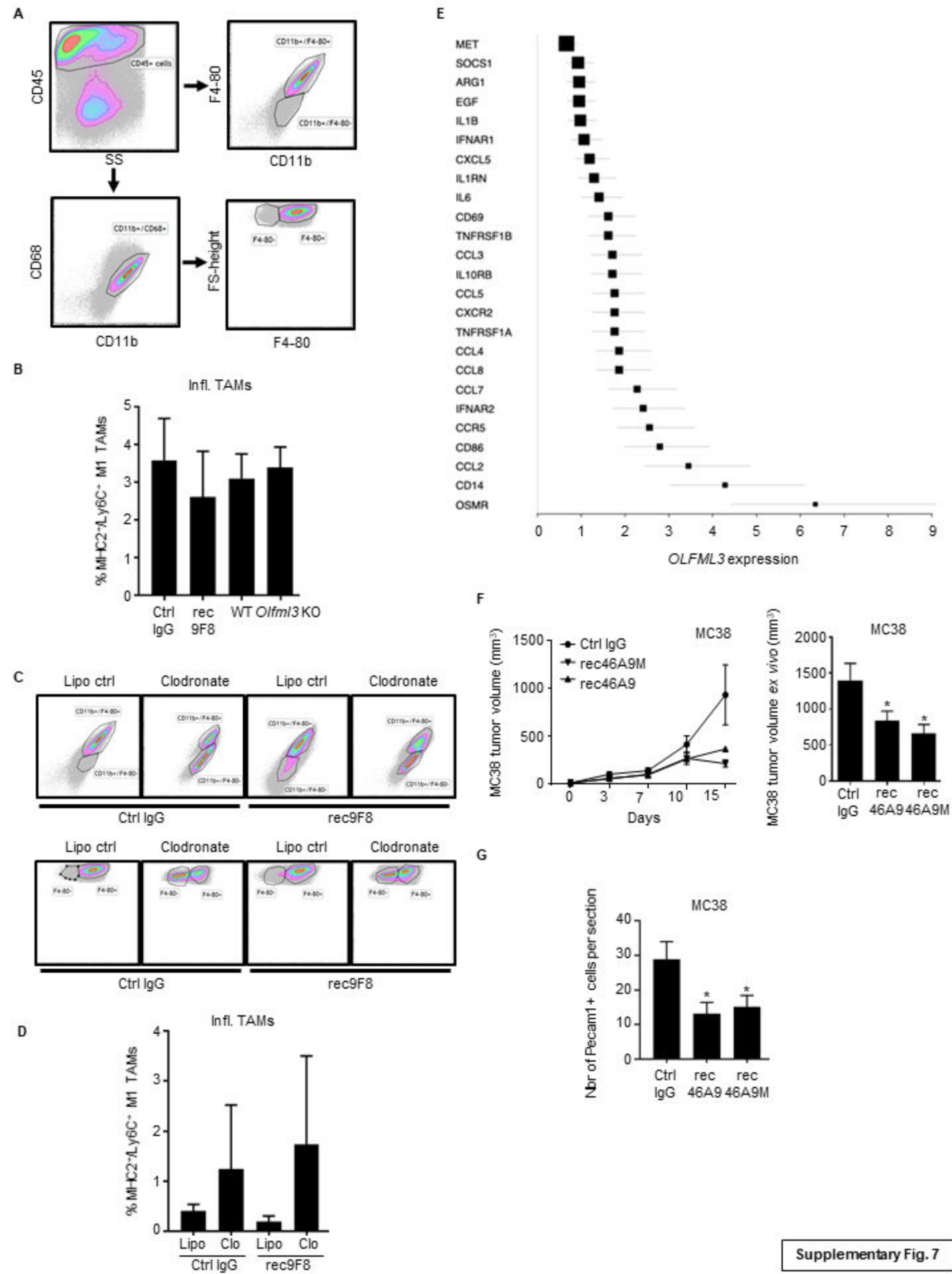

Supplement: Supplementary file 1 [file cancers-13-04625-s001.zip › Figures and Supplementary documents/Supplementary Figures/Figure S7.pdf]

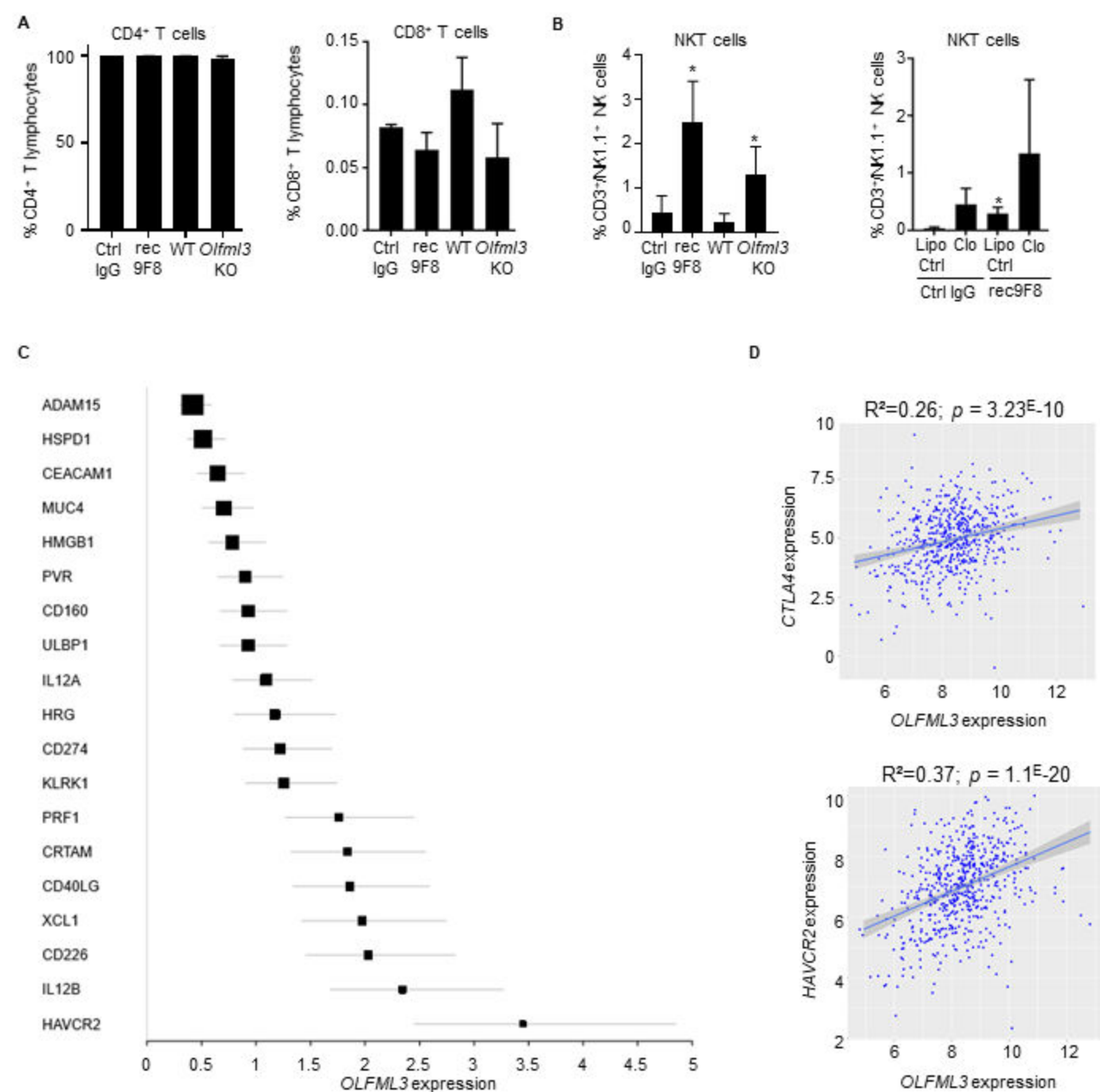

Supplement: Supplementary file 1 [file cancers-13-04625-s001.zip › Figures and Supplementary documents/Supplementary Figures/Figure S8.pdf]
